# Supplementary material for: Contrast limited adaptive histogram equalization (CLAHE) and colour difference histogram (CDH) feature merging capsule network (CCFMCapsNet) for complex image recognition
Source: PLoS One. 2025 Oct 31;20(10):e0335393. doi: 10.1371/journal.pone.0335393 (PMC12578171; doi:10.1371/journal.pone.0335393)
Supplement: Attachment — Submitted filename: Response to Reviewers.docx [file pone.0335393.s002.docx]

**Original Manuscript ID:** PONE-D-25-27327

**Original Article Title: “**Contrast Limited Adaptive Histogram Equalization (CLAHE) and Colour Difference Histogram (CDH) Feature Merging Capsule Network (CCFMCapsNet) for Complex image recognition”

**To:** PLOS ONE

**Re:** Response to Reviewers

Dear Editor,

We would like to express our profound gratitude to the Editor and the reviewers for their professional and constructive comments on our manuscript, “Contrast Limited Adaptive Histogram Equalization (CLAHE) and Colour Difference Histogram (CDH) Feature Merging Capsule Network (CCFMCapsNet) for Complex image recognition” *(ID:* PONE-D-25-27327). We have revised the manuscript according to the comments and suggestions of the academic Editor and the reviewers, and have responded to the academic editor's and the reviewers’ concerns. The responses to the reviewer’s comments are therefore attached.

Thank you for allowing a resubmission of our manuscript, with an opportunity to address the academic editor and the reviewers’ comments.

We are uploading (a) our rebuttal letter that responds to each of the comment (below) (Response to Reviewers), (b) an updated marked-up manuscript with yellow and green highlighting indicating changes made as suggested by Reviewer #1 and Reviewer #2 respectively ('Revised Manuscript with Track Changes), and (c) an unmarked, updated manuscript without highlights (Manuscript). Finally, the authors would like to affirm your office that the manuscript can be assigned to the same reviewers. For further information, please feel free to contact the corresponding author. His personal information is as follows:

**Mailing address:**	Mr. Steve Okyere-Gyamfi

Department of Computing and Information Sciences,

Catholic University of Ghana,

P. O. Box 363, Fiapre - Sunyani, Ghana

**Phone number:**	+233 (0) 263299901

**Email:**	steve.og@cug.edu.gh

Best regards

Steve Okyere-Gyamfi et al.

Reviewer #1: This manuscript introduces CCFM-CapsNet, an enhanced Capsule Network model integrating CLAHE and CDH to improve image classification in plant disease detection and standard benchmarks. While the proposed architecture shows promising results, the manuscript has several issues. Substantial revisions are required for the manuscript.

**Reviewer #1, Comment # 1:** In Section 3.5 (Datasets), the class label numbering for each dataset (e.g., “3,2,1,0” for apple, grape, corn) is inconsistent and sometimes confusing. The order of class labels changes between datasets, and explanations of which number corresponds to which class are not always clear. I recommend unifying the labeling convention across datasets and presenting the mappings between class numbers and disease categories in a clear table or standardized list.

**Response:** Thank you for pointing out the inconsistency. We have revised Section 3.5 to standardize the labeling format across all datasets. **Table I** has been added, clearly mapping each dataset’s class labels to their corresponding disease or categories. This enhances readability and clarity throughout the revised manuscript.	**[see page: 13-15]**

**Author action:**

**3.5 Datasets**

Eleven independent datasets (i.e., nine plant disease datasets from Kaggle, Mendley, etc, and two benchmark datasets) were used independently to run and assess the original and CCFM-CapsNets effectiveness, and compared with various cutting-edge CapsNet models found in the literature that used the same datasets to evaluate their performance. This includes the Apple, Corn, Grape, Pepper, Tomato, Potato, Banana, Mango, Rice, Fashion-MNIST, and CIFAR-10 datasets.

The datasets of ***Corn***, ***Apple***, ***Pepper***, ***Tomato, Grape,*** and ***Potato*** were sized 256x256, and are included in the Plant Village dataset (34).

***Banana:*** Consists of varying dimensions ranging from *2230x4000 to 3120x4208* (35).

***Mango:*** Consists of varying dimensions ranging from 240x240 to 320x240 (36).

***Rice:*** Consists of varying dimensions ranging from 216x289 to 344x516 (37).

***Fashion-MNIST:*** Consists of grayscale images, of size 28x28, and is complex (38).

***CIFAR-10:*** consists of 32x32x3. They have different backgrounds and are more complex than Fashion-MNIST (39).

Table I presents the total image count for each of the 11 datasets, along with the number of classes and the sample size per class.

Most of the datasets for plant disease are significantly imbalanced, with images that are very similar to each other and backgrounds that are not uniform. The pre-processing step utilized to these datasets was rescaling the images to a dimension of 32 x 32 x 3 to maintain manageability, except for the Fashion-MNIST dataset. Additionally, an 80:20 split technique was employed.

Table I: Summary of the 11 independent datasets used separately for model training and evaluation

| S/N | Dataset | Total no. of images | No. of Classes | Classes and no. of samples | Number of samples (images) |
| --- | --- | --- | --- | --- | --- |
| 1 | Apple | 3,171 | 4 | 0: Apple_scab | 630 |
|  |  |  |  | 1: Black_rot | 621 |
|  |  |  |  | 2: Cedar_apple_rust | 275 |
|  |  |  |  | 3: healthy | 1645 |
| 2 | Grape | 4,062 | 4 | 0: Black_rot | 1180 |
|  |  |  |  | 1: Esca_(Black_Measles) | 1383 |
|  |  |  |  | 2: Leaf_blight_(Isariopsis_Leaf_Spot) | 1076 |
|  |  |  |  | 3: healthy | 423 |
| 3 | Corn | 3,852 | 4 | 0: Cercospora_leaf_spot, Gray_leaf_spot | 513 |
|  |  |  |  | 1: Common_rust | 1192 |
|  |  |  |  | 2: Northern_Leaf_Blight | 985 |
|  |  |  |  | 3: healthy | 1162 |
| 4 | Pepper | 2,475 | 2 | 0: healthy | 1478 |
|  |  |  |  | 1: Bacterial_spot | 997 |
| 5 | Potato | 2,152 | 3 | 0: Healthy | 152 |
|  |  |  |  | 1: Early_blight | 1000 |
|  |  |  |  | 2: Late_blight | 1000 |
| 6 | Tomato | 18,160 | 10 | 0: Bacterial_spot | 2127 |
|  |  |  |  | 1: Early_blight | 1000 |
|  |  |  |  | 2: Late_blight | 1909 |
|  |  |  |  | 3: Leaf_Mold | 952 |
|  |  |  |  | 4: Septoria_leaf_spot | 1771 |
|  |  |  |  | 5: Spider_mites Two-spotted_spider_mite | 1676 |
|  |  |  |  | 6: Target_Spot | 1404 |
|  |  |  |  | 7: Tomato_mosaic_virus | 373 |
|  |  |  |  | 8: Tomato_Yellow_Leaf_Curl_Virus | 5357 |
|  |  |  |  | 9: Healthy | 1591 |
| 7 | Banana | 937 | 4 | 0: Cordana | 162 |
|  |  |  |  | 1: Pestalotiopsis | 173 |
|  |  |  |  | 2: Sigatoka | 473 |
|  |  |  |  | 3: Healthy | 129 |
| 8 | Mango | 4,000 | 8 | 0: Anthracnose | 500 |
|  |  |  |  | 1: Bacterial Canker | 500 |
|  |  |  |  | 2: Cutting Weevil | 500 |
|  |  |  |  | 3: Die Back | 500 |
|  |  |  |  | 4: Gall Midge | 500 |
|  |  |  |  | 5: Powdery Mildew | 500 |
|  |  |  |  | 6: Powdery Mildew | 500 |
|  |  |  |  | 7: Healthy | 500 |
| 9 | Rice | 5,932 | 4 | 0: Bacterial_blight | 1584 |
|  |  |  |  | 1: Blast | 1440 |
|  |  |  |  | 2: Brown_spot | 1600 |
|  |  |  |  | 3: Tungro | 1308 |
| 10 | Fashion-MNIST | 70,000 | 10 | 0: T-shirt/top | 7000 |
|  |  |  |  | 1: Trouser | 7000 |
|  |  |  |  | 2: Pullover | 7000 |
|  |  |  |  | 3: Dress | 7000 |
|  |  |  |  | 4: Coat | 7000 |
|  |  |  |  | 5: Sandal | 7000 |
|  |  |  |  | 6: Shirt | 7000 |
|  |  |  |  | 7: Sneaker | 7000 |
|  |  |  |  | 8: Bag | 7000 |
|  |  |  |  | 9: Ankle boot | 7000 |
| 11 | CIFAR-10 | 60,000 | 10 | 0: airplane | 6000 |
|  |  |  |  | 1: automobile | 6000 |
|  |  |  |  | 2: bird | 6000 |
|  |  |  |  | 3: cat | 6000 |
|  |  |  |  | 4: deer | 6000 |
|  |  |  |  | 5: dog | 6000 |
|  |  |  |  | 6: frog | 6000 |
|  |  |  |  | 7: horse | 6000 |
|  |  |  |  | 8: ship | 6000 |
|  |  |  |  | 9: truck | 6000 |

**Reviewer #1, Comment # 2:** While the manuscript provides many architectural details, the training procedures lack clarity in important aspects: there is no hyperparameter tuning processes, or evaluation protocols for train/validation/test splits beyond the 80:20 mention. I recommend providing a more thorough, step-by-step description of the experimental pipeline.

**Response:** We thank the reviewer for the valuable feedback regarding the clarity of our training procedures. In response, we have expanded the description of our experimental pipeline in the revised manuscript to provide a more thorough and step-by-step explanation of the hyperparameter tuning processes, the experimental pipeline, and the evaluation metrics used. **[see page: 15-18]**

**Author action:**

3.6 **Experimental setup**

**3.6.1 Hyperparameter Tuning**:
The following hyperparameters were chosen for performance evaluation of both the original and CCFM-CapsNetss training and testing, and compared with various cutting-edge CapsNet models found in the literature that used the same datasets to assess their performance:

- Learning rate: 0.001 with exponential decay (decay rate = 0.9)
- Batch size: 100
- Epochs: 200
- Routing iterations: 3
- Reconstruction loss coefficient (λ): 0.392
- All images in the various datasets were resized to 32X32X3, except Fashion-MNIST, which was 28x28x1
- For each of the 11 independent datasets used in this study, we applied an 80:20 train-test split. All splits were stratified to maintain class distribution.

**[see page: 15-16]**

**3.6.2 Experimental Pipeline**:

- All model training and testing took place in Keras, running on top of TensorFlow, using a 64-bit Windows computer equipped with an NVIDIA GeForce RTX 2080 SUPER GPU (8 GB).
- Training was performed separately for each of the 11 datasets using the same pipeline to ensure fair comparisons.
- The top-performing model was saved and used for evaluation.
- The original CapsNet implementation from Xifeng Guo from Sabour was used as the base and modified to accommodate the proposed architectural enhancements.
- Evaluation metrics included precision, accuracy, sensitivity or recall, F1-score, specificity, ROC-AUC, and PR-AUC.

**[see page: 16]**

**3.7 Performance Evaluation Measures**

The study employed a variety of metrics to assess classification performance:

- **Confusion Matrix:** Offers a comprehensive summary of both accurate and inaccurate predictions, enabling the computation of metrics such as sensitivity (recall), precision, specificity, F1-Score, and accuracy from TP-true positive, FP-false positive, TN-true negative, FN-false negative.
- **Validation Accuracy:** indicates the ratio of samples correctly classified compared to the overall number of instances. The overall validation accuracy reported reflects outcomes across all experiments, as shown in equation 11.

$$Accuracy= \frac{TP+TN}{TP+FP+TN+FN} [11]$$

- **Loss:** Measures how far the model’s predictions deviate from the true labels. Margin loss was specifically used during assessment.
- **Precision (P):** Measure of how many of the predicted positives are truly positives. This is shown in equation 12.

$$Precision\left( P \right)=\frac{TP}{TP+FP} [12]$$

- **Recall (R) / Sensitivity:** Measures how many of the actual positive instances are correctly detected by the model. This is shown in equation 13.

$$\frac{Recall\left( R \right)}{Sensitivity}=\frac{TP}{TP+FN} [13]$$

- **Specificity:** The proportion of true negatives identified among all actual negatives. This is shown in equation 14.

$$Specificity=\frac{TN}{TN+FP} [14]$$

- **F1-Score:** Combines precision and recall through their harmonic mean, ensuring a trade-off between them. This is shown in equation 15.

$$F1-Score=2\left( \frac{P*R}{P+R} \right) [15]$$

- **Area Under the Curve (AUC):** Performance evaluation of models are assessed with Receiver Operating Characteristic (ROC) and Precision–Recall (PR) curves, which are especially informative for imbalanced datasets. Larger AUC values signify a strong discriminative capability.
- **Clustering Analysis:** To gain insight into the feature distribution, t-SNE was used to visualize and interpret class capsule clusters generated by the model.
- **Model Complexity:** The study also measured performance by analyzing computational efficiency, expressed through parameter count and memory usage.

**[see page: 16-17]**

**Reviewer #1, Comment # 3:** The manuscript compares CCFM-CapsNet primarily with traditional and modified CapsNet models but does not include comparisons with recent state-of-the-art non-CapsNet architectures (e.g., ViT, EfficientNet). Including such benchmarks or at least a discussion of their relevance would significantly strengthen your claims about the proposed model’s performance.

**Response:** Thank you for the suggestion. While our study’s main focus is on enhancing CapsNet, we agree that at least a discussion of the relevance of recent state-of-the-art non-CapsNet architectures (e.g., ViT, EfficientNet) would significantly strengthen claims of the proposed model’s performance. Hence a discussion of such recent state-of-the-art non-CapsNet architectures have been discussed in the introduction section of the revised manuscript.	**[see page: 3-4]**

**Author action:**

Recent advances in image classification (especially in crop disease detection) have been dominated by CNNs such as EfficientNet and ResNet, DenseNet, as well as transformer-based models like the Vision Transformer (ViT) (3)(4)(5)(6)(7)(8). These models have also demonstrated impressive performance across large-scale benchmark datasets such as CIFAR-10, fashion-MNIST, ImageNet, etc., often surpassing human-level accuracy (9). Their success is largely attributable to hierarchical feature extraction (in CNNs) and global self-attention mechanisms (in ViTs), both of which enable strong generalization when large volumes of labeled data are available. For example, EfficientNet employs a joint scaling strategy to proportionally scale the width, depth, and resolution, yielding top-tier accuracy (5). Similarly, ViT leverages patch embeddings and multi-head attention to capture long-range dependencies, pushing the boundaries of image classification performance (6).

While these models are highly influential in advancing the field, they also highlight the gaps that Capsule Networks (CapsNets) are designed to address. CNNs and ViTs often struggle with preserving spatial hierarchies and viewpoint equivariance, which can be critical in fine-grained recognition tasks or cases with limited data, such as those in health, agriculture, etc. (10)(11).

As CNNs go deeper, they need broad data coverage to prevent overfitting (12). They also maintain good performance as the model depth increases; however, this introduces challenges, including significant parameter numbers, amplified complexity, increased memory requirements, and intensive computing requirements. To ensure CNNs and ViTs perform effectively and generalize well to new data, time-consuming and labour-intensive data augmentation techniques are necessary(12).

CapsNets, through their routing-by-agreement mechanism, are inherently designed to encode part–whole relationships, making them more interpretable and potentially more robust in low-data regimes addressing the limitations of CNN’s (11).

Capsules need less training data, are less impacted by class imbalance, and are more resilient to changes in spatial orientation. Despite these benefits of CapsNets, they also have some disadvantages (1). Due to the "crowding" problem, their performance is poor on complex images that have diverse backgrounds(12). This issue affects various datasets such as CIFAR-100, CIFAR-10, wild plant images, medical images, multilabel images, and many more. The Capsule network's sensitivity to the image background makes it prone to misclassification.

Additionally, CapsNet attempts to account for every detail in the image. Due to these properties, the network's performance can decline when processing detailed infected images. The encoder network's ineffective ability to extract features considerably hinders CapsNet's performance (13) (14). Consequently, enhancing the current capsule network algorithm is necessary to effectively classify these images.

To enhance the categorization of plant ailments employing CapsNet, we incorporated CapsNet with dynamic routing (11). Additionally, we introduced CDH in conjunction with CapsNet to extract crucial features for the first time. Moreover, we integrated CLAHE to improve visual clarity by reducing inherent noise, thereby enhancing the feature extraction capabilities of the encoder network.

It is worth highlighting that the current investigation is devoted to the advancement of CapsNet architectures rather than proposing a direct replacement for CNNs or ViTs. The comparisons in this study are therefore made against established CapsNet variants to ensure that the proposed contributions are evaluated fairly within the same model family. Nonetheless, discussing CNN- and transformer-based models remains relevant because they are part of the cutting-edge techniques in image classification. Their strengths and limitations provide a useful backdrop for understanding why improving CapsNets is significant. In particular, CNNs and ViTs, together with their variants are known to require extensive data augmentation and large-scale training datasets to generalize well (6)(7)(8), whereas CapsNets offer a principled approach to capturing spatial hierarchies with potentially fewer samples (10)(11). Thus, by improving CapsNet performance, our work contributes toward bridging the gap between biologically inspired models and the efficiency of mainstream architectures.

While CNN-based models and ViTs set the benchmark for large-scale classification performance, the relevance of CapsNets lies in their unique ability to capture pose and spatial relationships in a way that conventional models cannot and its ability to perform well on smaller datasets. The proposed CCFM-CapsNet therefore extends the applicability of CapsNet-based models, demonstrating that with appropriate architectural refinements, CapsNets can achieve competitive or superior performance suitable for resource-constrained devices, particularly in domains like agriculture and health, where interpretability, robustness, and data efficiency are critical.

**Reviewer #2:**

**Reviewer #2, Comment # 1:** What measures you took to ensure that model is not overfitted?

**Response:** Thank you for the suggestion. We now clarify in the revised manuscript the steps taken to prevent overfitting.	Several steps were taken to prevent overfitting:

1. The CLAHE preprocessing component also improved generalization by standardizing local contrast, reducing overfitting due to lighting variability.
2. We incorporated dropout layers into the CCFM-CapsNet architecture to mitigate overfitting.
3. We also reduced the primary capsule channels from 32 of the original CapsNet to 16, which also lowered the parameter count and computational cost, mitigated overfitting.

**[see page: 11-13]**

**Author action:**

1. CLAHE preprocessing component also improves generalization by standardizing local contrast and reducing overfitting due to lighting variability.

**[see page:11]**

CLAHE is employed to boost image contrast and restrict the neighbouring pixel procedure to diminish noise, improve image quality, and mitigate overfitting.	**[see page:12]**

2. a dropout layer to randomly deactivate neurons during training to help reduce the model’s reliance on individual neurons to improve robustness, promote generalization on unseen data, and help prevent overfitting.	**[see page:12]**
3. The concatenated feature map is then sent through a dropout layer with a batch normalizer, then through the Primary Capsule layer having 16 channels with 8 dimensions instead of 32 dimensions to help lower the parameter count, computational cost, and also mitigate overfitting, a kernel of size 3x3 and a stride of 2.	**[see page:13]**

**Reviewer #2, Comment # 2:** Perform k-fold validation, with minimum 10 Folds.

**Response:** We appreciate the reviewer’s suggestion to perform 10-fold cross-validation.

From the way we presented the datasets, it was confusing, and it confused the reviewers into thinking that we used the entire PlantVillage dataset at once to run the model, as indicated by reviewer# 1 comment #1 and reviewer #2 comment #4 (the paper cited used the entire PlantVillage dataset at once to run the model; DOI: 10.1134/S1054661825700087).

Therefore, the dataset section has been improved for better clarity and understanding as to the fact that, the 11 independent datasets (i.e. 9 plant disease datasets from Kaggle, Mendley, etc and 2 benchmark datasets-Fashion-MNIST and CIFAR-10 datasets), apple, grape, corn, pepper, potato, tomato, banana, mango, rice, Fashion-MNIST, and CIFAR-10 datasets, were used independently, as shown in Table I. These 11 independent datasets were trained and tested separately on the models to assess their performance and robustness, and the proposed CCFM-CapsNet performed comparably well on all the 11 different datasets, indicating its robustness and generalizability.

We choose to use 11 independent datasets to train and test the performance and robustness of the models, as solid support in the machine learning and deep learning literature shows that evaluating a model across multiple diverse datasets is often a stronger indication of robustness and generalizability for several critical reasons;

**1. Diverse datasets as a test of robustness**
Evaluating a model across a wide range of datasets provides a more realistic assessment of robustness than repeated splits of a single dataset. Performance on a single, repeatedly used dataset can give a misleading impression of generalization, since the model is only exposed to variations within that dataset. In contrast, testing on additional, independently constructed datasets better reflects real-world conditions, where data distributions often differ (Recht et al., 2019).

**2. Generalization beyond similar distributions**
Cross-validation is valuable for estimating generalization *within* a dataset, but it largely tests a model’s ability to perform on data drawn from the *same distribution*. Evaluating on multiple independent datasets, however, assesses whether a model can generalize to *different distributions*, which is crucial for real-world robustness. For instance, in natural language processing, the GLUE benchmark uses nine separate datasets to test generalization across diverse tasks, demonstrating the strength of evaluating on multiple datasets rather than relying solely on internal cross-validation (Wang et al., 2018).

**3. Insights from meta-analyses and benchmark design**
Broad evaluation across multiple datasets is essential to capture the general behavior of algorithms. Koh et al. (2021) showed that evaluating deep learning models across diverse datasets provides deeper insights into their stability, limitations, and generalization capacity than evaluations confined to a single dataset.

**Application in this study**
Following this principle, we employed **11 independent datasets**—9 plant disease datasets (apple, grape, corn, pepper, potato, tomato, banana, mango, rice) sourced from Kaggle, Mendeley, and related repositories, together with 2 benchmark datasets (Fashion-MNIST and CIFAR-10). Each dataset was used independently for training and testing, as summarized in Table I. This approach provides a comprehensive and rigorous evaluation of model performance and robustness across varying data domains and distributions, strengthening the validity of the proposed architecture.

**References**

- Recht, B., Roelofs, R., Schmidt, L., & Shankar, V. (2019). *Do ImageNet classifiers generalize to ImageNet?* International Conference on Machine Learning (ICML), 5389–5400.
- Wang, A., Singh, A., Michael, J., Hill, F., Levy, O., & Bowman, S. R. (2018). *GLUE: A multi-task benchmark and analysis platform for natural language understanding*. arXiv preprint arXiv:1804.07461.
- Koh, P. W., Sagawa, S., Marklund, H., Xie, S. M., Zhang, M., Balsubramani, A., ... & Liang, P. (2021). *WILDS: A benchmark of in-the-wild distribution shifts*. Proceedings of the International Conference on Machine Learning (ICML), 5637–5664.

**Reviewer #2, Comment # 3:** Improve image quality, few images are very blur and hard to understand.

**Response:** Thank you for this observation. We have reviewed and improved the few blurry images among the figures.

**Reviewer #2, Comment # 4:** DOI: 10.1134/S1054661825700087, also claimed more than 99% accuracy on Plant-Village dataset. This is the same dataset which you used in the article. How your research is more promising then the above mentioned.

**Response:** Thank you for the review.

This paper (DOI: 10.1134/S1054661825700087) used the entire plant village dataset as one dataset and had the accuracy > 99%. On the other hand, our model did not use the plant village dataset as one dataset, some plant datasets like apple, grape, corn, pepper, potato, tomato, were obtained from the plant village dataset and used independently as individual datasets, then banana, mango, rice, Fashion-MNIST, and CIFAR-10 datasets were also acquired separately from other sources and also used independently making 11 independent datasets which all of them were trained and tested independently using the original and the proposed CCFM-CapsNet model.

Abstract of the paper whose DOI was provided is shown below showing the datasets that was used.

### Abstract (DOI: 10.1134/S1054661825700087)

The development of an automatic system for classifying plants and their diseases at different stages of growth could play an important role in both increasing crop yields and assisting in the care of indoor plants. However, existing studies on plant and disease recognition are not systematic enough and use different datasets, making it difficult to identify the best models. In this paper, we consider the problem of constructing benchmarks for the problem of simultaneous classification of plants and their diseases and evaluate the performance of three models, MobileNetV3Small, EfficientNetB0, and DenseNet121, **pretrained on the ImageNet and further trained on the PlantVillage and PlantDoc datasets.** As a result of the experiments, it was found that the EfficientNetV2B0 model was the most effective for the task of plant disease recognition with an **accuracy of 0.997 on the PlantVillage dataset and 0.96 on the PlantDoc dataset.**

Reference

Shiyan, A.S., Kozlov, I.D., Baimuratov, I.R. *et al.* Recognizing Plants and Their Diseases: Benchmarks for Multiclass and Multilabel Classification. *Pattern Recognit. Image Anal.* 35, 159–168 (2025). https://doi.org/10.1134/S1054661825700087
